# Preclinical Evidence for the Efficacy of CD79b Immunotherapy in B-cell Precursor Acute Lymphoblastic Leukemia

**DOI:** 10.1097/HS9.0000000000000754

**Published:** 2022-07-15

**Authors:** Lennart Lenk, Dorothee Winterberg, Fotini Vogiatzi, Anna Laqua, Lea Spory, Ahmad Mayar, Anna Dietterle, Gina Münch, Christian Vokuhl, Julia Richter, Andrew G. Polson, Thomas Schüler, Ulf D. Kahlert, Matthias Peipp, Thomas Valerius, Martin Schrappe, Gunnar Cario, Hassan Jumaa, Elias Hobeika, Monika Brüggemann, Ameera Alsadeq, Denis M. Schewe

**Affiliations:** 1 Department of Pediatrics I, ALL-BFM Study Group, Christian-Albrechts University Kiel and University Medical Center Schleswig-Holstein, Kiel, Germany; 2 Department of Medicine II, University Hospital Schleswig-Holstein, Kiel, Germany; 3 Department of Pathology, Section of Pediatric Pathology, University Hospital Bonn, Germany; 4 Department of Pathology, Hematopathology Section and Lymph Node Registry, Christian-Albrechts University Kiel and University Medical Center Schleswig-Holstein, Kiel, Germany; 5 Genentech Research and Early Development, San Francisco, CA, USA; 6 Institute of Molecular and Clinical Immunology, Medical Faculty, Otto-Von-Guericke University Magdeburg, Germany; 7 Molecular and Experimental Surgery, Clinic for General, Visceral, Vascular, and Transplant Surgery, Medical Faculty, University Hospital Magdeburg, Germany; 8 Department of Medicine II, Division of Antibody-Based Immunotherapy, Christian-Albrechts University Kiel and University Medical Center Schleswig-Holstein, Kiel, Germany; 9 Department of Medicine II, Section for Stem Cell Transplantation and Immunotherapy, Christian-Albrechts University Kiel and University Medical Center Schleswig-Holstein, Kiel, Germany; 10 Institute of Immunology, Ulm University Medical Center, Ulm, Germany; 11 Department of Pediatrics, Otto-von-Guericke-University, Magdeburg, Germany

Patients with B-cell precursor acute lymphoblastic leukemia (BCP-ALL) have a favorable prognosis. However, current treatment protocols are based on intensive cytotoxic chemotherapy and therapy options are limited when patients relapse.^1^ Hence, there is an urgent need for novel immunotherapy approaches.

A potential novel target could be the pre-B-cell receptor (pre-BCR) signaling complex for which an integral role in B-cell malignancies has been proposed.^2^ The pre-BCR consists of the µ heavy chain (µHC), the surrogate light chain (VpreB and Lambda 5), and a signaling heterodimer composed of CD79a (Igα) and CD79b (Igβ). Activation of the pre-BCR induces downstream signaling via phosphorylation of tyrosine residues within the immunoreceptor tyrosine-based activation motifs (ITAMs) of the cytoplasmic tails of CD79a/CD79b.^3^ Due to its high abundance on mature B cells, the BCR complex has become an important target for diffuse large B-cell lymphoma (DLBCL) treatment and the CD79b antibody drug conjugate (ADC) Polatuzumab Vedotin (PolVed) has shown therapeutic efficacy in DLBCL-frontline treatment.^4^

Nevertheless, the CD79a/CD79b heterodimer is already expressed at the pro-B-cell stage before a productive immunoglobulin gene rearrangement is accomplished, even without associated µHC.^5^ Moreover, we recently reported that the pre-BCR signaling unit CD79a is crucial for BCP-ALL engraftment in vivo, particularly in the central nervous system (CNS), in BCR-ABL1^+^ and E2A-PBX1^+^ patient-derived xenograft (PDX) models.^6^ We therefore hypothesized that CD79b may also serve as a therapeutic target in BCP-ALL.

Here, we show that surface (s)CD79b is expressed in different subgroups of pediatric BCP-ALL patients and that targeting CD79b with a monoclonal antibody reduced CNS involvement of sCD79b positive (sCD79b^+^) PDX samples in vivo. Moreover, the CD79b-ADC PolVed significantly diminished overall leukemia burden and prolonged mouse survival in sCD79b^+^ BCP-ALL-PDX models of different cytogenetic backgrounds.

First, to test whether CD79b is important for leukemic engraftment, we applied a murine/murine transplantation model as described previously.^6^ Murine precursor B cells harboring a truncated cytoplasmic tail of CD79b, which results in the loss of the ITAM and therefore functional CD79b^7^ (referred to as CD79b-ITAM-KO) were malignantly transformed by stable transduction with a *BCR-ABL1*-construct (Suppl. Figure S1A–B). Transformed CD79b-ITAM-KO cells showed a similar proliferation pattern as control (Ctr) cells in vitro (Suppl. Figure S1C). However, upon transplantation into NOD scid gamma (NSG) mice, Ctr cells exposed profound leukemia engraftment in the spleen (Sp; *P* = 0.0079), bone marrow (BM, *P* = 0.0079), and CNS (*P* = 0.0079) leading to sacrifice of all Ctr animals after 25 days while animals injected with CD79b-ITAM-KO cells did not show signs of leukemia at that timepoint (Figure 1A–C, Suppl. Figure S1D). An additional group of mice transplanted with CD79b-ITAM-KO cells was left for survival analysis and mice of this group were still free of leukemia upon termination of the experiment after 162 days (Figure 1C, Suppl. Figure S1E–F) indicating that CD79b is required for leukemia engraftment in vivo.

**Figure 1.**
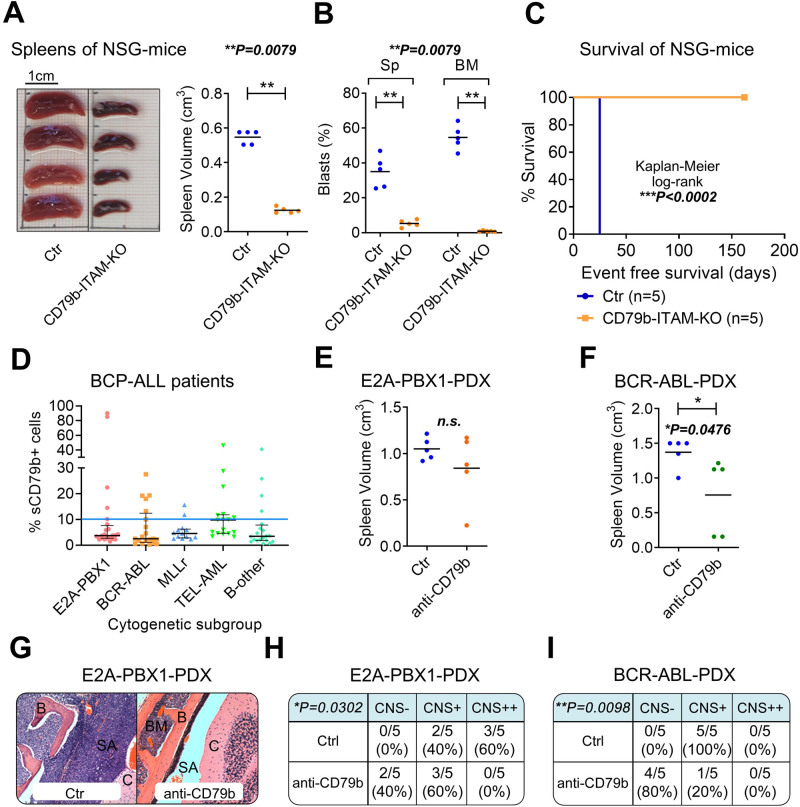
**CD79b is important for BCP-ALL engraftment in vivo.** CD79b is important for BCP-ALL engraftment in vivo: (A–C) Precursor B cells isolated from either wildtype BALB/c mice or mice carrying a truncated variant of CD79b (CD79b-ITAM-KO) were malignantly transformed with *BCR-ABL1*. Ctr and CD79b-ITAM-KO cells were injected into NSG mice (n = 5 Ctr, n = 10 CD79b-ITAM-KO). To examine niche specific engraftment, animals injected with CD79b-ITAM-KO cells (n = 5) were sacrificed when the mice injected with Ctr cells (n = 5) showed signs of overt leukemia (such as ataxia, splenomegaly, weight loss, or >70% leukemic cells in the peripheral blood; all Ctr animals had developed overt leukemia by day 25). One group of mice injected with CD79b-ITAM-KO (n = 5) was maintained for survival analysis. (A), Sp volumes (unpaired *t* test), and (B) percentage of ALL cells in Sp and BM were measured (unpaired, 2-sided *t* test). (C), Differences in mouse survival of animals injected with Ctr cells versus CD79b-ITAM-KO cells were determined using Kaplan-Meier log-rank statistics. The experiment was terminated after 162 d and mice bearing CD79b-ITAM-KO cells sacrificed without showing signs of overt leukemia. (D), Diagnostic BM or blood samples of BCP-ALL patients of different cytogenetic backgrounds were analyzed for surface (s)CD79b expression within the CD45^dim^/CD19^+^ BCP-ALL cell population. The blue line depicts the cutoff of 10% sCD79b^+^ cells. Positivity for CD79b was detected in 5/24 E2A-PBX1+, 5/20 BCR-ABL+, 2/13 MLLr, 7/17 TEL-AML1+, and 4/20 B-other BCP-ALL patients. (E–I), NSG mice were transplanted with BCP-ALL-PDX cells from an E2A-PBX1^+^ and a BCR-ABL^+^ patient and treated with a monoclonal blocking antibody against CD79b (anti-CD79b, 1 mg/kg) or a Ctr vehicle (n = 5, respectively) starting the day after injection, modeling an MRD situation (intravenous treatment on day +1, +3, +7, +14 and every 14 d thereafter as described previously^8^). Animals were sacrificed when the first mouse showed signs of overt leukemia and (E–F) volumes of extracted Sp (indicator for leukemic engraftment) were measured, unpaired 2-sided *t* test. (G), Representative hematoxylin/eosin-stained histology sections of NSG mouse heads of Ctr and anti-CD79b treated E2A-PBX1^+^ PDX mice, red lines indicate blast-filled areas (B = bone, BM = bone marrow, C = cerebellum; Ctr = control; SA = subarachnoid space). (H–I), CNS infiltration was assessed by semi-quantitative scoring as described previously,^6,8^ Fisher exact test. BCP-ALL = B-cell precursor acute lymphoblastic leukemia; CNS = central nervous system; MLLr = MLL rearranged; MRD = minimal residual disease; n.s. = not significant; sCD79b^+^ = sCD79b positive; Sp = spleen.

To identify patients who may benefit from CD79b-immunotherapy, we investigated the frequency of sCD79b expression in BCP-ALL patients. We measured sCD79b levels via flow cytometry in diagnostic BM/blood samples of pediatric BCP-ALL patients of different cytogenetics (gating strategy depicted in Suppl. Figure S2). Using a cutoff of ≥10% sCD79b^+^ BCP-ALL cells (according to AEIOP-BFM guidelines^9^), we detected sCD79b-positivity in 23 of 94 (24%) patient samples (Figure 1D). Interestingly, a population of sCD79b^+^ cases was detected in all subgroups investigated, including E2A-PBX1^+^ (5/24), BCR-ABL^+^ (5/20), MLL rearranged (MLLr) (2/13), TEL-AML1^+^ (7/17), and B-other (4/20) BCP-ALL patients.

Due to the lack of BCP-ALL engraftment of transformed CD79b-ITAM-KO cells, we hypothesized that blocking CD79b with a monoclonal antibody reduces BCP-ALL engraftment in vivo. To test this hypothesis, we applied an unconjugated CD79b-IgG1-antibody (clone SN8, anti-CD79b) on NSG mice bearing either E2A-PBX1^+^ or BCR-ABL1^+^ PDX samples (Suppl. Table S1/PDX1-2) with high sCD79b expression (53.6% sCD79b^+^ cells and 25.9% sCD79b^+^ cells, respectively, Suppl. Figure S3, Suppl. Figure S4A–B). Animals were injected with 1 × 10^5^ PDX cells and anti-CD79b-treatment was initiated 1 day post-injection (“minimal residual disease [MRD] model”^8,10,11^). All animals were sacrificed when the first mouse showed clinical signs of leukemia. Anti-CD79b-immunotherapy resulted in a small reduction of leukemia burden in the Sp and BM of E2A-PBX1^+^ PDX mice and a significant reduction of Sp and BM engraftment in BCR-ABL^+^ PDX mice (Figure 1E and F, Suppl. Figure S4C–F). Of note, anti-CD79b-treatment promoted a significant reduction of CNS involvement in both PDX models (*P* = 0.0302 and *P* = 0.0098, respectively; Figure 1G–I) indicating that CD79b blockade impacts the engraftment of BCP-ALL cells in vivo and survival of BCP-ALL cells in the CNS.

Next, we investigated if CD79b-immunotherapy using a CD79b-ADC would outperform the efficacy of CD79b blockade. Hence, we performed the same in vivo experiment applying PolVed therapy in our sCD79b^+^ PDX-MRD models (Suppl. Table S1/PDX1-2). Indeed, PolVed therapy led to a profound anti-leukemic effect in both PDX models showing significantly reduced Sp sizes as well as blast numbers in Sp and BM as compared with Ctr animals (*P* < 0.0001, respectively; Figure 2A and B; Suppl. Figure S5A–B). Accordingly, all PolVed-treated PDX mice were CNS negative upon sacrifice of Ctr animals (*P* = 0.0079, respectively; Figure 2C and D). Analysis of further PolVed-treated animals (n = 5), which were left for survival analysis, showed a significant survival prolongation under PolVed therapy in both PDX models (median overall survival [MOS] 106 d versus not definable; *P* = 0.0027 and 76 versus 148 d; *P* = 0.0027; Figure 2E and F). Of note, 4/5 PolVed-treated E2A-PBX1^+^ PDX mice were free of BCP-ALL-PDX cells upon termination of the experiment after 236 days (Figure 2E, Suppl. Figure S5C).

**Figure 2.**
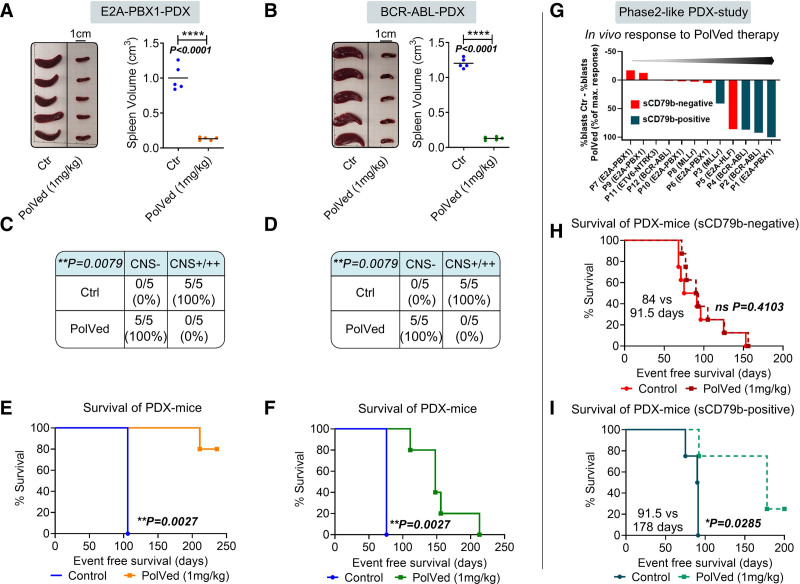
**PolVed shows preclinical efficacy in sCD79b positive PDX samples in vivo.** PolVed shows preclinical efficacy in sCD79b positive PDX samples in vivo: (A–F) NSG mice were transplanted with BCP-ALL-PDX cells from an E2A-PBX1^+^ and a BCR-ABL^+^ patient and treated with the CD79b-ADC PolVed (1 mg/kg, n = 10) or a Ctr vehicle (n = 5) starting the day after injection, modeling an MRD situation (intravenous treatment on day +1, +3, +7, +14 and every 14 d thereafter as described previously^8^). Five animals, respectively, were sacrificed when the first mouse showed signs of overt leukemia (such as ataxia, splenomegaly, weight loss, or >70% leukemic cells in the PB; all Ctr animals had developed overt leukemia at this time point). One group of mice treated with PolVed (n = 5) was maintained for survival analysis. (A and B), Volumes of extracted spleens (indicator for leukemic engraftment) were measured, unpaired 2-sided *t* test. (C and D), CNS infiltration was assessed by semi-quantitative scoring as described previously,^6,8^ Fisher exact test. (E and F), Therapy-associated differences in the survival of NSG mice bearing E2A-PBX1^+^ or BCR-ABL^+^ BCP-ALL cells were determined using Kaplan-Meier log-rank statistics. The experiment was terminated after 236 d and 4/5 BCR-ABL^+^ PDX mice treated with PolVed were sacrificed without showing signs overt leukemia. (G–I), A phase 2-like PDX study was performed using sCD79b-positive (≥10% sCD79b^+^ cells, n = 4), and CD79b-negative (<10% sCD79b^+^ cells, n = 8) PDX samples from different cytogenetic subgroups (5xE2A-PBX1^+^, 3xBCR-ABL^+^, 2xMLLr, 1xE2A-HLF^+^, and 1xETV6-NTRK3^+^). Two NSG mice per patient were injected with PDX cells, randomly assigned into treatment groups and PolVed therapy was initiated upon detection of 1% PDX cells in the PB, modeling an overt leukemia situation. (G), Blood of both, Ctr and PolVed treated animals bearing the same PDX sample was withdrawn when one of the 2 PDX mice showed signs of overt leukemia and the number of hCD45^+^/hCD19^+^/mCD45^–^ cells in the PB was measured via flow cytometry. The waterfall plot shows the difference in PB blasts between respective Ctr and PolVed treated mice normalized to the maximum blast reduction (sorted from weakest therapy response to highest therapy response). Animals not showing clinical signs of overt leukemia or >70% PB blasts at this timepoint received further treatment until reaching termination criteria. Therapy-associated differences in the survival of NSG mice bearing (H) sCD79b^–^ and (I) sCD79b^+^ PDX cells were determined using Kaplan-Meier log-rank statistics. ADC = antibody drug conjugate; BCP-ALL = B-cell precursor acute lymphoblastic leukemia; BCP-ALL-PDX = B-cell precursor acute lymphoblastic leukemia-patient-derived xenograft; CNS = central nervous system; Ctr = control; hCD45+/hCD19+/mCD45– = human (h)CD45+hCD19+(murine) mCD45-; MLLr = MLL rearranged; MRD = minimal residual disease; ns = not significant; NSG = NOD scid gamma; PB = peripheral blood; PDX = patient-derived xenograft; PolVed = Polatuzumab Vedotin.

To further validate the target-specificity of PolVed in sCD79b^+^ BCP-ALL cells, we compared the efficacy of PolVed to that of the CD30-ADC Brentuximab Vedotin (BreVed) in vivo in the E2A-PBX1^+^ sCD79b^+^/CD30^–^ PDX model (Suppl. Figure S6A, Suppl. Table S1/PDX1). PolVed had anti-leukemic efficacy and BreVed treatment resulted in Sp sizes and blast counts in Sp and BM comparable with that of Ctr animals suggesting that PolVed kills BCP-ALL cells in a target-specific manner (Suppl. Figure S6B–C).

Finally, to test the efficacy of PolVed treatment in a broad range of BCP-ALL samples, we performed a phase 2-like PDX study^10–12^ using sCD79b^+^ (≥10% sCD79b^+^ cells, n = 4) and CD79b^–^ (<10% sCD79b^+^ cells, n = 8) PDX samples from different cytogenetic subgroups (5xE2A-PBX1^+^, 3xBCR-ABL^+^, 2xMLLr, 1xE2A-HLF^+^, and 1xETV6-NTRK3^+^, Suppl. Table S1/PDX1-12). Two NSG mice per patient were injected with PDX cells, randomly assigned into groups and PolVed therapy was initiated upon detection of 1% PDX cells in the peripheral blood, modeling overt leukemia (Suppl. Figure S7).^10–12^ When one of the 2 mice (Ctr or PolVed therapy) bearing the same PDX-sample developed clinical signs of overt leukemia, the peripheral blood of both PDX mice was analyzed for the number of BCP-ALL cells. This analysis showed a distinct blast reduction in 4/4 sCD79b^+^, but only 1/8 sCD79b^–^ PDX samples in vivo upon PolVed therapy (Figure 2G, Suppl. Table S1). Moreover, survival analysis revealed that whereas Ctr and PolVed-treated sCD79b^–^ animals showed comparable median survival times (MOS = 84 versus 91.5 d; Figure 2H), PolVed treatment led to a significant survival prolongation in sCD79b^+^ PDX mice (MOS = 91.5 versus 178 d; *P* = 0.0285; Figure 2I). This group included 1xE2A-PBX1^+^, 2xBCR-ABL^+^, and 1xMLLr PDX models (Suppl. Table S1). Interestingly, 1 sCD79b^–^ PDX-sample exposing a distinct population of sCD79b^+^ cells (6.8% sCD79b^+^ cells, E2A-HLF^+^ BCP-ALL) displayed substantial response and survival prolongation upon PolVed therapy (Figure 2G, Suppl. Table S1/PDX5). These data indicate that PolVed-immunotherapy may be effective in sCD79b^+^ cases of different BCP-ALL subgroups.

Antibody-based immunotherapies such as Blinatumomab have become an important tool in BCP-ALL treatment. Yet, the observation of tumor immune-escape via downregulation of the target-antigen, for example, CD19 motivates the identification of novel immunotherapy targets.^13^ We show the presence of CD79b on the surface of diagnostic patient samples of different BCP-ALL subgroups. This is particularly interesting as previous reports suggested that only certain cytogenetic subgroups such as E2A-PBX1^+^ BCP-ALL are considered as pre-BCR positive, whereas most BCP-ALL cases, including BCR-ABL^+^ BCP-ALL do not express µHC and therefore a functional pre-BCR on the cell surface.^14^ Our data promote the view that CD79b is expressed on the surface of BCP-ALL cells irrespective of a fully assembled pre-BCR signaling complex as previously hypothesized.^6^ This is further supported by the recent finding that the pre-BCR surrogate light chain component VpreB was detected in subpopulations of BCP-ALL patient samples regardless of cytogenetic subgroups.^15^ Yet, the role of CD79b and VpreB (and other BCR components) may differ markedly in BCP-ALL. Unlike VpreB, CD79b harbors an ITAM in the cytoplasmic domain by which CD79b on the cell surface may promote downstream signaling, irrespective of a fully arranged pre-BCR complex, thereby enhancing the survival and proliferation of BCP-ALL cells. Accordingly, in our model CD79b deletion had a direct effect on ALL propagation in vivo, indicating a functional role in ALL-pathogenesis. Since CD79b immunotherapy has already entered clinical routine in other B-cell malignancies,^4^ PolVed therapy may represent an interesting treatment alternative for BCP-ALL, potentially also in relapsed/refractory disease. To this end, PolVed treatment was also effective in BCR-ABL^+^, MLLr and E2A-HLF^+^ PDX samples, which are considered high-risk subgroups. The prospective measurement of sCD79b+ in newly diagnosed and (CD19^–^) relapsed BCP-ALL patients may help to identify patients who could benefit from CD79b immunotherapy. Moreover, an important step before clinical transition will be to preclinically test the efficacy and tolerability of PolVed in combination with standard-of-care treatments using PDX models.^10,11^ In this respect, the efficacy of CD79b immunotherapy could be tested in comparison or combination with small molecule inhibitors targeting the pre-BCR signaling pathway.^14^ Overall, gaining a better understanding of the role of the various components of the pre-BCR in leukemia development and relapse may improve diagnostic and therapeutic options in BCP-ALL.

## ACKNOWLEDGMENTS

We thank the patients and physicians who contributed samples and data for this study. We thank Katrin Timm-Richert, Katrin Neumann, Gabriele Riesen, Birthe Fedders, and Silvia Iwersen for the excellent technical assistance.

## AUTHOR CONTRIBUTIONS

LL and DW designed and performed experiments and analyzed data. FV, AL, LS, AM, AD, GM, CV, JR, and AGP performed experiments and analyzed data. MS, GC, and MB provided ALL samples and clinical data. TS, UDK, MP, TV, and AGP discussed the research direction. EH and HJ provided mouse models. LL, AA, and DMS initiated and designed the study and discussed the research direction. LL and DMS conceived and wrote the article. All authors discussed the article.

## DISCLOSURES

LL received research funding from OSE Immunotherapeutics outside the submitted work. MB received consulting fees from PRMA Consulting, research funding from Amgen, honoraria from Novartis, Pfizer, and Amgen and was an advisory board member for Incyte and Amgen. DMS was an advisory board member for Bayer, SOBI, and Jazz Pharmaceuticals and received research funding from OSE Immunotherapeutics. MS received research funding from Shire, and from Servier, as well as fees for Advisory Board functions from Jazz Pharmaceuticals and Servier. AGP is an employee of Genentech. Polatuzumab Vedotin was provided by Genentech, San Francisco, CA. All the other authors have no conflicts of interest to disclose.

## SOURCES OF FUNDING

DMS is funded by the Deutsche Krebshilfe e. V. (111963), the Wilhelm Sander Stiftung (2016.110.1 and 2019.119.1), the Deutsche José-Carreras-Leukämiestiftung (DJCLS 17 R/2017), and the Deutsche Forschungsgemeinschaft (CRU5010; P6). HJ is supported by the Deutsche Krebshilfe and Deutsche Forschungsgemeinschaft (SFB1074; projects A10, B6) and European Research Council advanced grant. EH is supported by the Deutsche Krebshilfe and Deutsche Forschungsgemeinschaft (SFB1074; projects A9).

## METHODS

Detailed information on material and methods are given in the Supplementary Material.

## DATA AVAILABILITY STATEMENT

All data supporting the findings of this study are available within the article and its supplementary information files. No codes were used for data analysis.

## Supplementary Material


