# Supplementary material for: Preclinical Evidence for the Efficacy of CD79b Immunotherapy in B-cell Precursor Acute Lymphoblastic Leukemia
Source: Hemasphere. 2022 Jul 15;6(8):e754. doi: 10.1097/HS9.0000000000000754 (PMC9351922; doi:10.1097/HS9.0000000000000754)
Supplement: Supplementary file 1 [file hs9-6-e754-s001.docx]

**Supplementary Data**

**Supplementary methods**

**Establishment of murine leukemia cells**

Bone marrow (BM)-cells from WT or CD79b^Δ^ ^1^ mice were cultured for 3 to 7 days in Iscove’s medium (Biochrom AG) containing 10% heat-inactivated FCS (Sigma-Aldrich), 2 mM l-glutamine, 100 U/ml penicillin (Gibco), 100 U/ml streptomycin (Gibco), and 50 µM 2-mercaptoethanol. The medium was supplemented in excess with the supernatant of J558L plasmacytoma-cells stably transfected with a vector encoding murine IL7. Then, pro-B-cells were retrovirally transformed with either an empty pMIG vector or with a pMIG vector expressing BCR-ABL1. Transformed cells were selected by IL7 withdrawal and kept in optimum conditions ^2^. One million BCR-ABL1 transformed cells were then injected intravenously into NSG-mice and animals sacrificed when the first mouse showed signs of overt leukemia. One group was left over for survival analysis (Supplementary Figure 2A).

**Expression assays**

Total RNA was isolated using Direct-zol™ RNA Kit (Zymo Research) or ReliaPrep™ RNA Cell Miniprep System (Promega), and synthesis of cDNA was performed with the RevertAid First Strand cDNA Synthesis Kit (Thermofisher) as mentioned previously ^3^. Quantitative PCR (qRT-PCR) analyses were performed on ABI7900HT PCR machine (Applied Biosystems) using Fast SYBR Green Master Mix (Applied Biosystems). For detection of CD79b, the QIAGEN Quantitect assay #QT00203651 was used. Quantitative-PCR analyses were performed as published previously ^3^ employing the delta-delta CT method ^4^.

**Patient samples**

94 BCP-ALL patients of different cytogenetics were treated according to ALL-Berlin-Frankfurt-Münster (BFM) 2000 or 2009 protocols. Informed consent was obtained in accordance with the Declaration of Helsinki. The study was approved by the ethical committee of the Christian-Albrechts University Kiel (D 437/17).

**Flow Cytometry**

At least 1x10^6^ diagnostic BM/blood samples (ficollized, cryopreserved) or patient derived xenograft (PDX)-cells (ficollized, cryopreserved or fresh) were washed with PBS and subjected to fixable viability stainings using the Zombie Aqua Fixable Viability Kit (Biolegend). Cells were washed in FACS-Buffer and incubated with primary antibody cocktail for 30 min at 8°C for surface staining. Permeabilization for intracellular staining was performed using the Cytofix/Cytoperm™ Fixation/Permeablization Kit (BD Biosciences). The cells were incubated with antibodies for intracellular stainings 30 min at 8°C. The following antibodies were used for flow cytometry:

| Target | Structure | Origin | Clone | Dye | Refence | Company |
| --- | --- | --- | --- | --- | --- | --- |
| hCD79b | cytoplasm | mouse | SN8 | PE | 335833 | BD |
| hCD79a | cytoplasm | mouse | HM47 | APC | 333505 | Biolegend |
| hCD79b | surface | mouse | SN8 | BB515 | 565225 | BD Horizon |
| hCD19 | surface | mouse | HIB19 | PE/Cy7 | 302216 | Biolegend |
| hCD19 | surface | mouse | HIB19 | PE | 302208 | Biolegend |
| hCD45 | surface | mouse | 2D1 | APC/H7 | 560274 | BD Pharmigen |
| hCD45 | surface | mouse | HI30 | FITC | 304006 | Biolegend |
| mCD45 | surface | rat | 30-F11 | APC | 17-0451-82 | Invitrogen |

Samples were directly analyzed using a MACSQuant X (Miltenyi Biotec) flow cytometer. A total of 100,000 events were collected from each sample when possible. The lymphocyte gate was analyzed depending on distinguished FSC vs. SSC properties. Singlets were then selected (FSC-A vs FSC-H). The living cells were identified using Zombie Aqua Fixable Viability dye and further analyzed according to their surface or intracellular protein stains. BCP-ALL-cells were identified by hCD19+/hCD45^dim^, normal B-cells by hCD19+/hCD45^high^ and T-cells by hCD19-/hCD45^high^ staining features. To indicate the boundaries between sCD79b, cyCD79b and cyCD79a negative and positive populations in BCP-ALL and B-cell populations of patient samples, gates were set according to T-cell populations (commonly sCD79b-/cyCD79b+/cyCD79a-) and an additional fluorescence-minus-one (FMO) control-stained probe, respectively, (Supplementary Figure 2). For PDX-samples, sCD79b, cyCD79b and cyCD79a positive populations were identified using an FMO control and additionally using hCD45^-^/hCD19^-^/mCD45^+^ cells as internal negative controls (Supplementary Figure 3). The sample preparations from different organs or patient samples acquired at different timepoints were treated in a comparable way and were acquired using the same settings. FlowJo v.10.7 was used for data analysis.

**BCP-ALL xenografts**

NOD.Cg-Prkdcscid Il2rgtm1Wjl/SzJ (NSG) mice were purchased from Charles River and xenografts generated in accordance with governmental regulations (Schleswig-Holstein Ministerium für Energiewende, Landwirtschaft, Umwelt, Natur und Digitalisierung) ^5–7^. One million ALL-cells were injected intravenously into female NSG-mice (6–10 weeks of age) and leukemic engraftment was followed by detection of human CD45+/murine CD45−/human CD19+ cells in the peripheral blood via flow cytometry analysis.

**Antibody treatment *in vivo***

NSG-mice were injected with 1 × 10^6^ BCP-ALL-cells/animal. Animals were treated intravenously on day +1, +3, +7, +21, +35, +48 and +56 with 1 mg/kg of anti-CD79b antibody (clone SN8, ANC-301-824), Polatuzumab Vedotin or Brentuximab Vedotin, as indicated in the figure legends. All mice of both treatment groups were sacrificed when the first mouse showed signs of overt leukemia (detection of >75% hCD19^+^/hCD45^+^/mCD45^-^ BCP-ALL cells in the peripheral blood or clinical signs of leukemia including loss of weight or activity, organomegaly, hind-limb paralysis). Mice were randomly allocated into each treatment group and no blinding was used. Leukemic infiltration of the murine CNS was assessed in histological sections in blinded experiments and the scorings CNS− (no CNS infiltration of the leptomeninges), CNS+ (week infiltration of the leptomeninges) and CNS++ (strong and multilayered meningeal infiltration) were discriminated ^5,7^.

**Statistical analysis**

Statistical analysis was performed using GraphPad PRISM 9.00, SPSS 22 and SigmaPlot 12.5. Gaussian distribution was tested using the Shapiro-Wilk test. In case of normal distribution, statistical significance was assessed using an unpaired t-test, otherwise the Mann-Whitney U test was applied. In case of normal distribution and multiple groups, ANOVA was used. Differences in survival were calculated using Kaplan-Meier log-rank statistics. A *P*-value of <0.05 was considered significant.

^
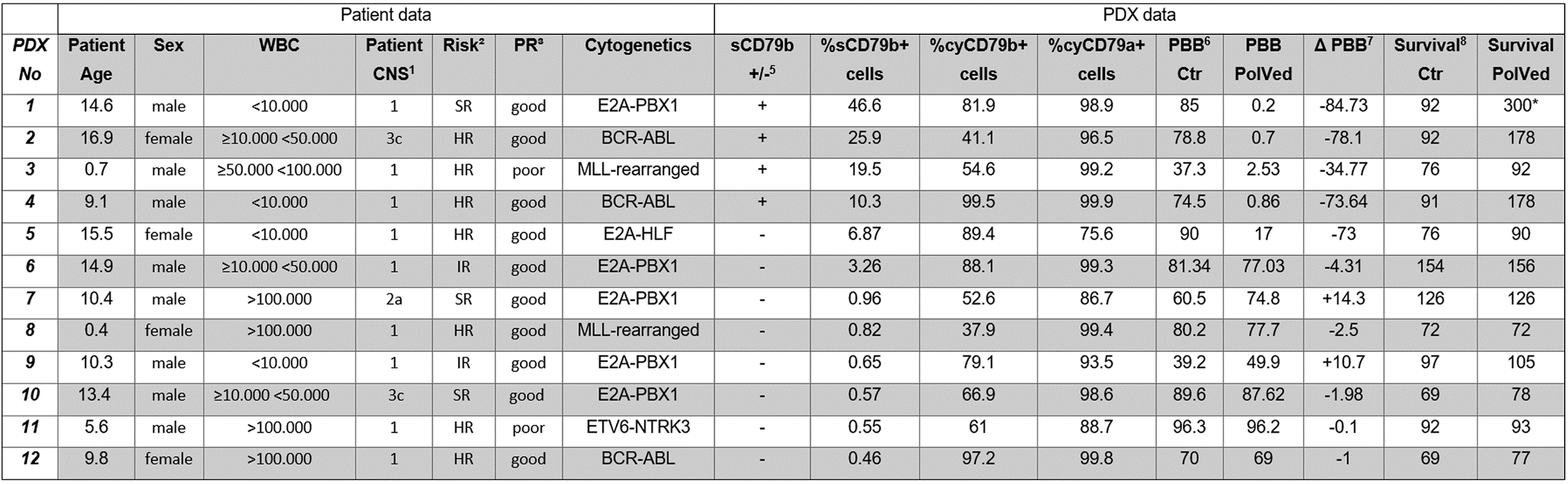
^**Supplementary Table 1: Patient characteristics for patient derived xenografts (PDX) used in *in vivo* experiments**

^1^Clinical definition of CNS status according to the ALL-BFM2009 protocol

CNS1: neither clinical nor radiological signs of CNS involvement AND no blasts in the cerebrospinal fluid (CSF) cytospin.

CNS2: neither clinical nor radiological signs of CNS involvement AND CNS2a: <10 per microliter red blood cells (RBC) and no macroscopic blood; ≤ 5 per microliter white blood cells (WBC); positive blasts in cytospin.

CNS2b: macroscopic blood and/or ≥ 10 per microliter RBC; ≤ 5 per microliter WBC; positive blasts in cytospin.

CNS2c: macroscopic blood and/or ≥ 10 per microliter RBC; >5 per microliter WBC; positive blasts in cytospin; negative according to algorithm (WBCL/RBCL)/(WBCB/RBCB) >2.

CNS3-CNS3a: <10 per microliter RBC and no macroscopic blood; >5 per microliter WBC; positive blasts in cytospin.

CNS3b: macroscopic blood and/or ≥ 10 per microliter RBC; >5 per microliter WBC; positive according to algorithm (WBCL/RBCL)/(WBCB/RBCB) >2.

CNS3c: clinical sings of CNS involvement. radiologically detectable cerebral lesion. retinal infiltrations.

^2^Risk stratification according to MRD risk groups: MRD-SR: TP1+2 negative. MRD-IR: TP1 and/or TP2 <10^-3^. MRD-HR: TP2 ≥ 10^-3^.

^3^PR: Prednisone response; G: good (less than 1000 leukemic blasts/µl blood on treatment day 8); P: poor (more than 1000/µl on day 8)

^4^CNS status determined via semi quantitative scoring as published ^5,8^.

N/A Data not available; WBC: White blood cell count at initial diagnosis

^5^Surface (s)CD79-positivity as determined by the number of sCD79b^+^/hCD45^+^/hCD19^+^/mCD45^-^ cells. <10% sCD79b^+^ BCP-ALL cells = negative (-); ≥10%-100% sCD79b^+^ BCP-ALL cells = positive (+).

^6^PBB: Peripheral blood blasts = Relative number of hCD45^+^/hCD19^+^/mCD45^-^ cells in the peripheral blood of control and PolVed-treated animals bearing the same PDX sample, obtained on the same day when one of the two animals showed signs of overt leukemia (experimental setup depicted in Supplementary Figure 7).

^7^Delta peripheral blood blasts = PBB Control animal – PBB PolVed treated animal

^8^Survival of respective control treated or PolVed treated PDX-mouse

**Supplementary Figures/ Figure legends**

**
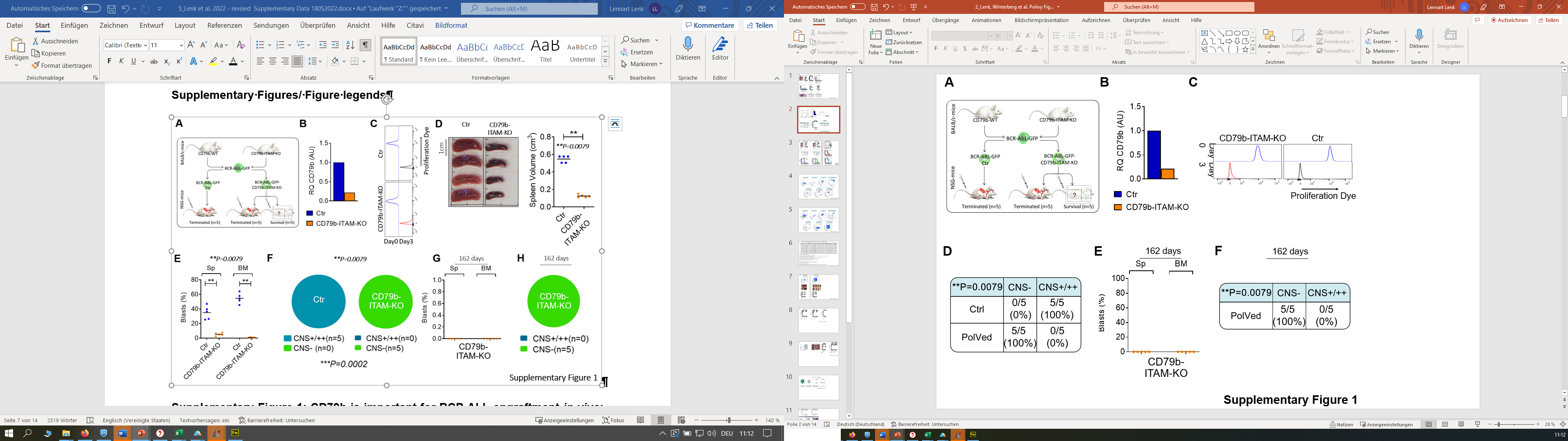
**

**Supplementary Figure 1: CD79b is important for BCP-ALL engraftment *in vivo*:** Precursor B-cells isolated from either wildtype BALB/c-mice or mice carrying a truncated variant of CD79b (CD79b-ITAM-KO) were malignantly transformed with BCR-ABL1. **A)** Experimental setup for the CD79b-knockdown-transplantation model. **B)** Downregulation of CD79b was validated via quantitative realtime-PCR. **C)** For determination of the *in vitro* proliferation of control (Ctr) or CD79b-ITAM-KO-cells, the cells were labelled with proliferation dye (eFluor 670) and cultured for 3 days. Cells were analyzed by flow cytometry directly after the labelling (day 0) and at the end of the experiment (day 3, representative graph out of 3 replicates shown). **D-H**) Ctr and CD79b-ITAM-KO-cells were injected into NSG-mice (n=5 Ctr, n=10 CD79b-ITAM-KO). To examine niche specific engraftment, animals injected with CD79b-ITAM-KO-cells (n=5) were sacrificed when the mice injected with Ctr-mice (n=5) showed signs of overt leukemia (such as ataxia, splenomegaly, weight loss or >70% leukemic cells in the peripheral blood). One group of mice injected with CD79b-ITAM-KO-cells (n=5) was maintained for survival analysis. (**D**) CNS infiltration was assessed by semi-quantitative scoring, Fisher’s exact test. (**E-F**) The experiment was terminated after 162 days. No animal injected with CD79b-ITAM-KO-cells exposed ALL cells in the (**E**) Sp, BM or (**F**) CNS at that time point.


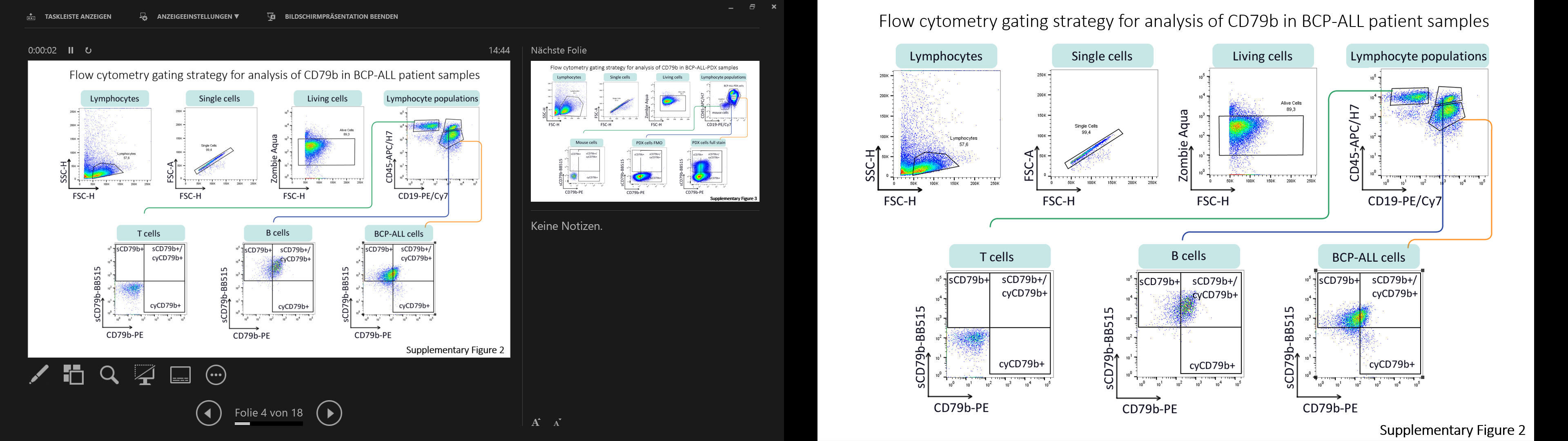


**Supplementary Figure 2:** **Flow cytometry gating strategy for analysis of CD79b in BCP-ALL patient samples:** One million cells of diagnostic bone marrow (BM) or blood samples from pediatric BCP-ALL patients of different cytogenetic backgrounds were analyzed per staining and 100,000 total events were collected from each sample when possible. The lymphocyte gate was analyzed depending on distinguished FSC vs. SSC properties. Singlets were then selected (FSC-A vs FSC-H). The living cells were identified using Zombie Aqua Fixable Viability dye and further analyzed according to their surface or intracellular protein stains. BCP-ALL-cells were identified by hCD19+/hCD45^dim^, normal B-cells by hCD19+/hCD45^high^ and T-cells by hCD19-/hCD45^high^ staining features. To indicate the boundaries between sCD79b, cyCD79b and cyCD79a negative and positive populations in BCP-ALL and B-cell populations, gates were set according to T-cell populations (commonly sCD79b-/cyCD79b+/cyCD79a-) and an additional fluorescence-minus-one (FMO, full staining without respective sCD79, cyCD79b and cyCD79a antibody) control-stained probe, respectively.


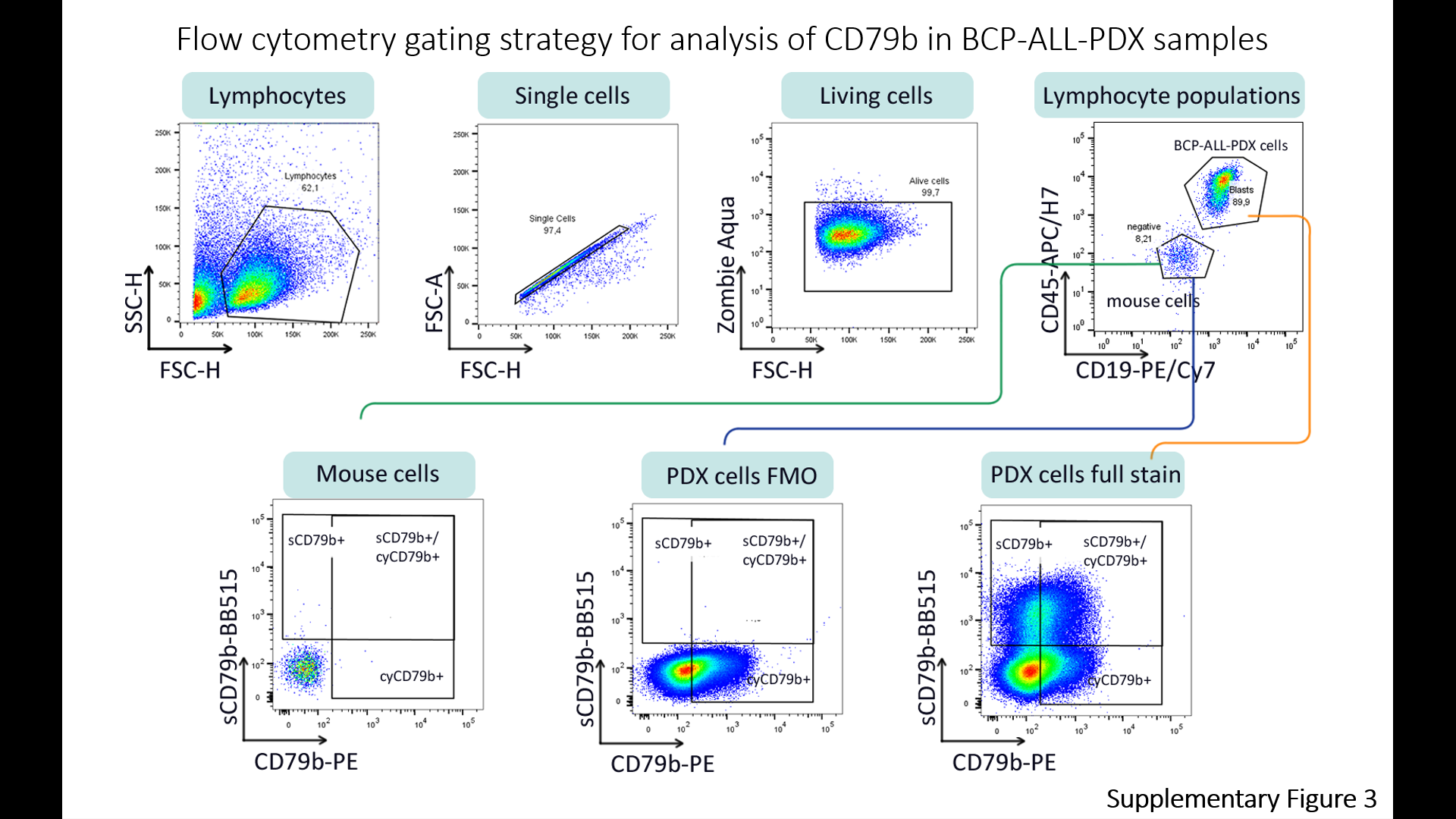


**Supplementary Figure 3:** **Flow cytometry gating strategy for analysis of CD79b in BCP-ALL patient derived xenograft (PDX)-samples:** One million cells from BCP-ALL-PDX-samples were analyzed per staining and 100,000 total events were collected from each sample when possible. The lymphocyte gate was analyzed depending on distinguished FSC vs. SSC properties. Singlets were then selected (FSC-A vs FSC-H). The living cells were identified using Zombie Aqua Fixable Viability dye and further analyzed according to their surface or intracellular protein stains. BCP-ALL-PDX-cells were identified by hCD19^+^/hCD45^+^/mCD45^-^ staining features. To indicate the boundaries between sCD79b, cyCD79b and cyCD79a negative and positive populations within the PDX-cell population, gates were set according to hCD19^-^/hCD45^-^/mCD45^+^ cell populations and an additional fluorescence-minus-one (FMO, full staining without respective sCD79b, cyCD79b and cyCD79a antibody) control-stained probe, respectively.


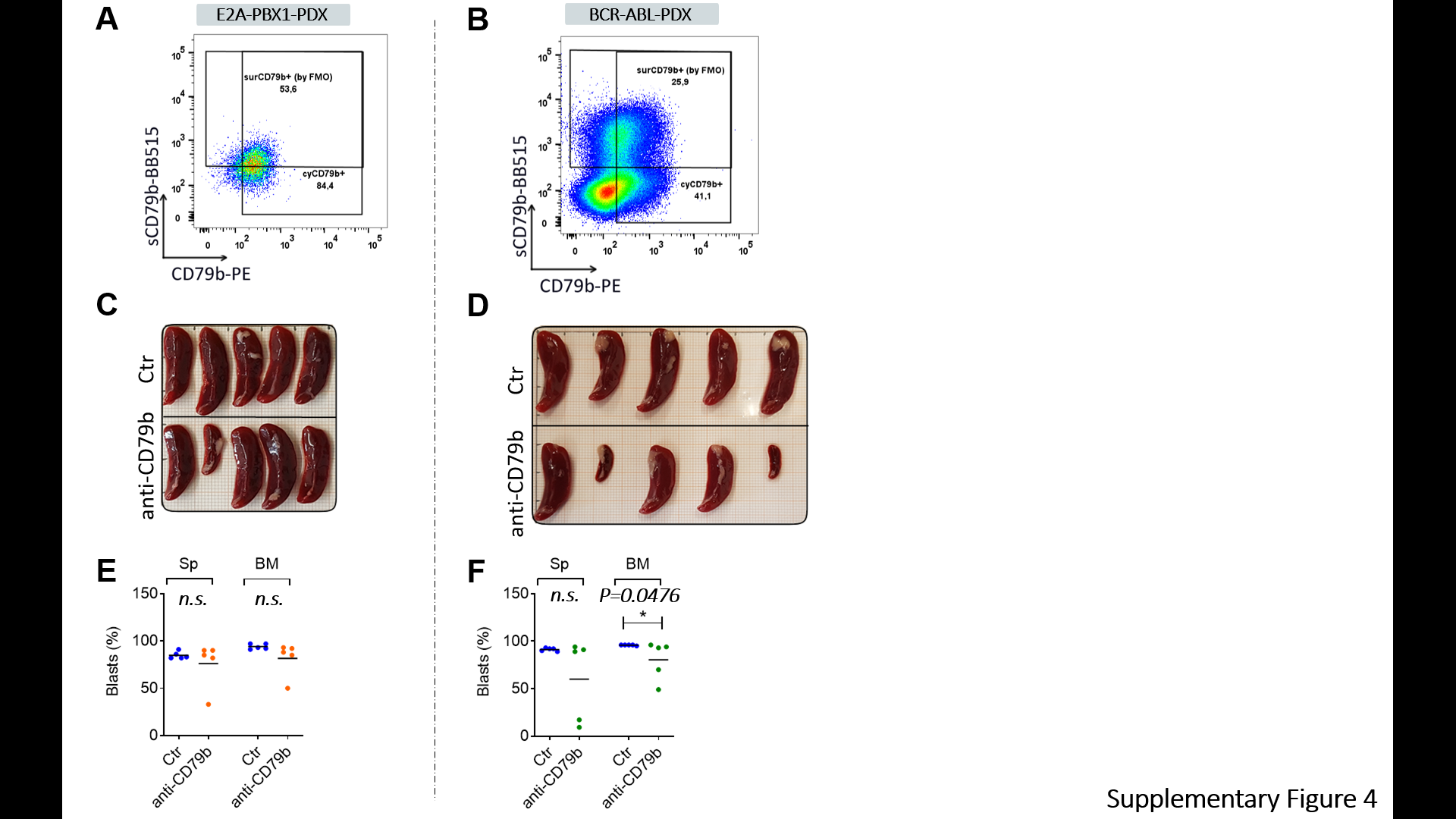


**Supplementary Figure 4:** **CD79b is important for BCP-ALL *in vivo* engraftment A-F**) NSG-mice were transplanted with BCP-ALL PDX-cells from an E2A-PBX1^+^ and a BCR-ABL^+^ patient and treated with an unconjugated CD79b-IgG1-antibody (clone SN8, anti-CD79b) or a control vehicle (Ctr)(n=5, respectively) starting the day after injection, modelling an MRD-situation (intravenous treatment on day +1, +3, +7, +14 and every 14 days thereafter as described previously ^5^). Animals were sacrificed when the first mouse showed signs of overt leukemia (such as ataxia, splenomegaly, weight loss or >70% leukemic cells in the peripheral blood). (**A-B**) Representative flow cytometry analysis of the PDX-samples depicting the relative number of sCD79b^+^/cyCD79b^+^ cells within the hCD45^+^/hCD19^+^ cell population. (**C-D**) Representative images of mouse spleens (Sp) after extraction as an indicator for leukemic engraftment *in vivo.* (**E-F**) The relative numbers of hCD45^+^/hCD19^+^/mCD45^-^ cells in the spleen and bone marrow (BM) were measured, unpaired two-sided t-test.

**
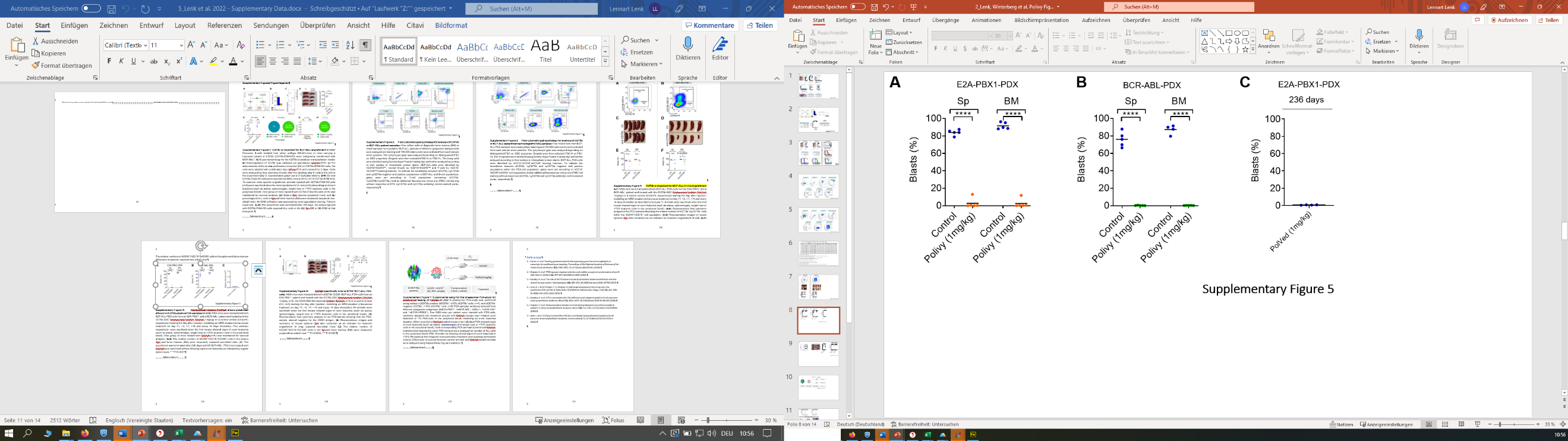
**

**Supplementary Figure 5:** **Polatuzumab Vedotin (PolVed) shows preclinical efficacy in CD79b-positive PDX-samples *in vivo*:** NSG-mice were transplanted with BCP-ALL-PDX-cells from an E2A-PBX1^+^ and a BCR-ABL^+^ patient and treated with the CD79b-ADC Polatuzumab Vedotin (PolVed, 1mg/kg) or a control vehicle (Ctr)(n=5, respectively) starting the day after injection, modelling an MRD-situation (intravenous treatment on day +1, +3, +7, +14 and every 14 days thereafter). Five animals, respectively, were sacrificed when the first mouse showed signs of overt leukemia (such as ataxia, splenomegaly, weight loss or >70% leukemic cells in the peripheral blood). One group of mice treated with PolVed (n=5) was maintained for survival analysis. (**A-B**) The relative number of hCD45^+^/hCD19^+^/mCD45^-^ cells in the spleen (Sp) and bone marrow (BM) were measured, unpaired two-sided t-test. (**C**) The experiment was terminated after 236 days and 4/5 BCR-ABL^+^ PDX-mice treated with PolVed were sacrificed without having developed overt leukemia at this timepoint indicated by the absence of detectable populations of hCD45^+^/hCD19^+^/mCD45^-^ cells. ****P<0.0001


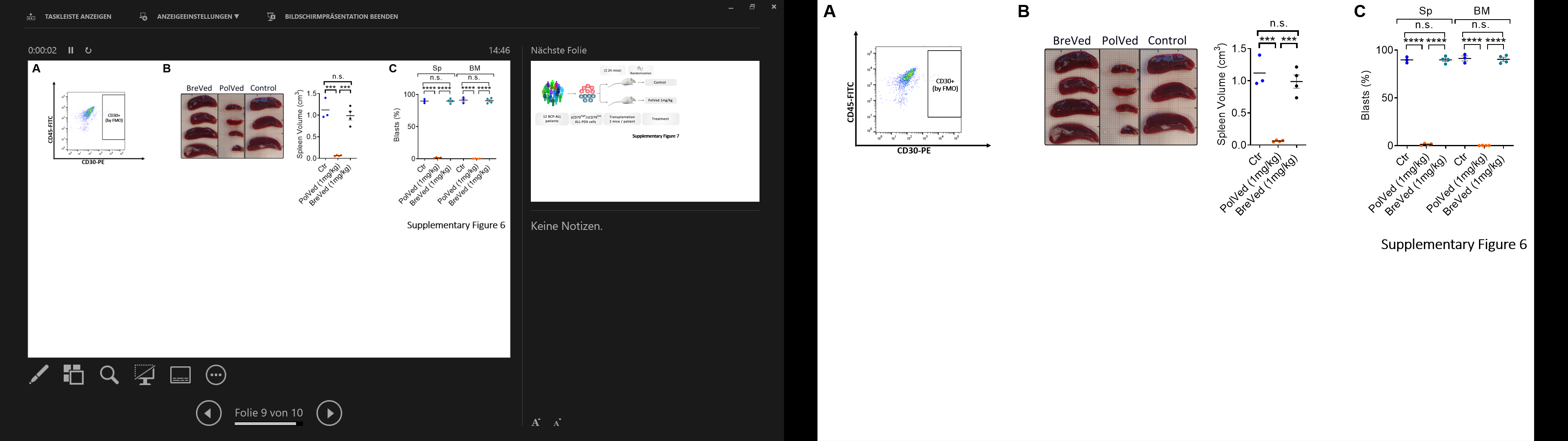


**Supplementary Figure 6:** **PolVed specifically kills sCD79b^+^ BCP-ALL-PDX-cells:** NSG-mice were transplanted with sCD79b^+^/CD30^-^ BCP-ALL-PDX-cells from an E2A-PBX1^+^ patient and treated with the CD79b-ADC Polatuzumab Vedotin (PolVed, 1mg/kg, n=5), the CD30-ADC Brentuximab Vedotin (BreVed, n=5) or a control vehicle (Ctr, n=3) starting the day after injection, modelling an MRD-situation (intravenous treatment on day +1, +3, +7, +14 and every 14 days thereafter). All animals were sacrificed when the first mouse showed signs of overt leukemia (such as ataxia, splenomegaly, weight loss or >70% leukemic cells in the peripheral blood). (**A**) Representative flow cytometry analysis of the PDX-sample showing that the PDX-sample stained negative for the CD30 antigen. (**B**) Representative images and volumetry of mouse spleens (Sp) after extraction as an indicator for leukemic engraftment *in vivo,* unpaired two-sided t-test*.* (**C)** The relative number of hCD45^+^/hCD19^+^/mCD45^-^ cells in the Sp and bone marrow (BM) were measured, unpaired two-sided t-test. ***P=0.0003, ****P<0.0001

**
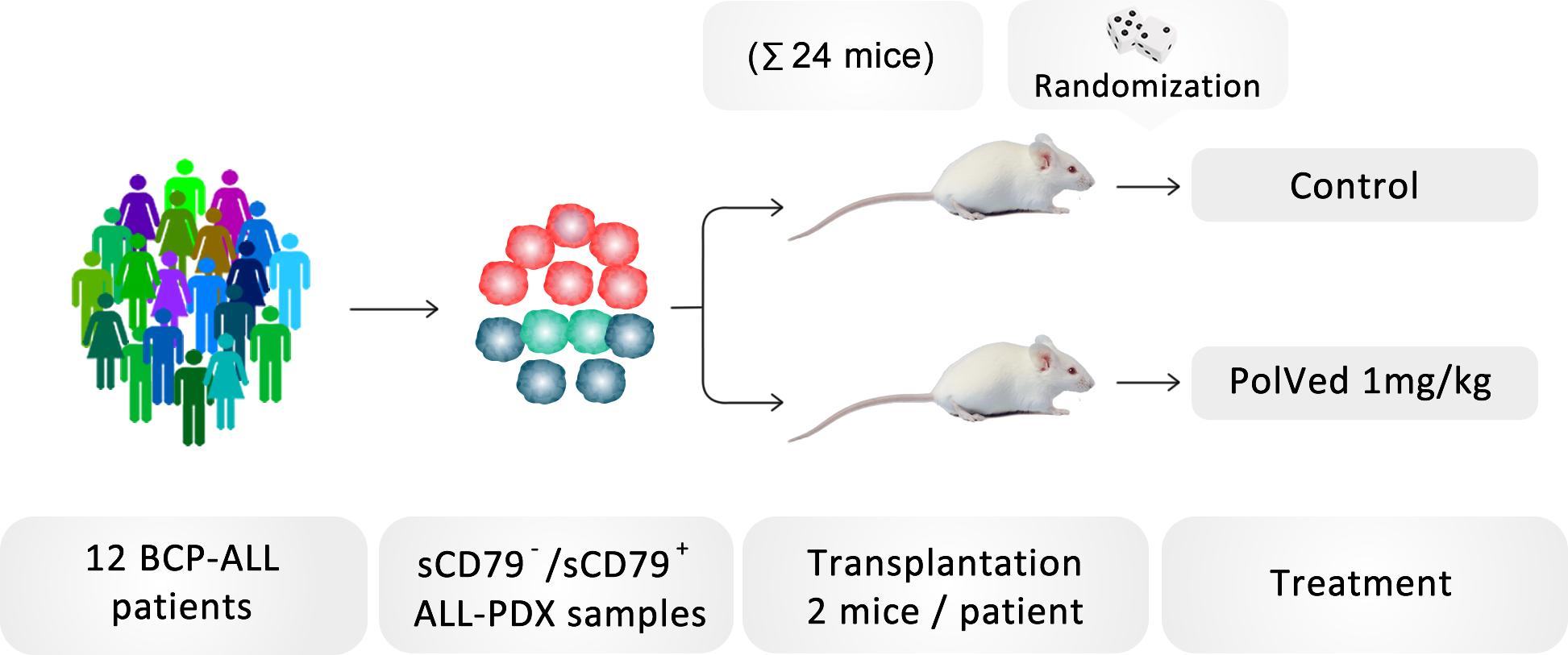
**

**Supplementary Figure 7: Experimental setup for the phase2-like PDX-study for preclincical testing of PolVed *in vivo*:** A phase2-like PDX-study was performed using surface (s)CD79b-positive (sCD79b^+^; ≥10% sCD79b^+^ cells, n=4), and sCD79b-negative (CD79b^-^; <10% sCD79b^+^ cells, n=8) PDX-samples randomly selected from different cytogenetic subgroups (5xE2A-PBX1^+^, 3xBCR-ABL^+^, 2xMLLr, 1xE2A-HLF^+^ and 1xETV6-NTRK3^+^). Two NSG-mice per patient were injected with PDX-cells, randomly assigned into treatment groups and PolVed therapy was initiated upon detection of 1% PDX-cells in the peripheral blood, modelling an overt leukemia situation. When a control or PolVed-treated mouse of an individual PDX showed signs of overt leukemia (such as ataxia, splenomegaly and weight loss or >70% leukemic cells in the peripheral blood), both corresponding PDX-animals (Control and PolVed-treated animal bearing the same PDX sample) were analyzed for number of ALL cells in the peripheral blood (PB). Animals not showing clinical signs of overt leukemia or >70% PB-blasts at this timepoint received further treatment until reaching termination criteria. Differences in survival between control animals and PolVed-treated animals were analyzed using Kaplan-Meier log-rank statistics.

References

1. Casola, S. *et al.* Tracking germinal center B cells expressing germ-line immunoglobulin γ1 transcripts by conditional gene targeting. *Proceedings of the National Academy of Sciences of the United States of America* **103,** 7396–7401; 10.1073/pnas.0602353103 (2006).

2. Shojaee, S. *et al.* PTEN opposes negative selection and enables oncogenic transformation of pre-B cells. *Nature medicine* **22,** 379–387; 10.1038/nm.4062 (2016).

3. Alsadeq, A. *et al.* The role of ZAP70 kinase in acute lymphoblastic leukemia infiltration into the central nervous system. *Haematologica* **102,** 346–355; 10.3324/haematol.2016.147744 (2017).

4. Livak, K. J. & Schmittgen, T. D. Analysis of relative gene expression data using real-time quantitative PCR and the 2(-Delta Delta C(T)) Method. *Methods (San Diego, Calif.)* **25,** 402–408; 10.1006/meth.2001.1262 (2001).

5. Alsadeq, A. *et al.* IL7R is associated with CNS infiltration and relapse in pediatric B-cell precursor acute lymphoblastic leukemia. *Blood* **132,** 1614–1617; 10.1182/blood-2018-04-844209 (2018).

6. Vogiatzi, F. *et al.* Daratumumab eradicates minimal residual disease in a preclinical model of pediatric T-cell acute lymphoblastic leukemia. *Blood* **134,** 713–716; 10.1182/blood.2019000904 (2019).

7. Lenk, L. *et al.* CD79a promotes CNS-infiltration and leukemia engraftment in pediatric B-cell precursor acute lymphoblastic leukemia. *Commun Biol* **4,** 73; 10.1038/s42003-020-01591-z (2021).
